# CRISPR-Cas9-Mediated Gene Editing in Hematological Disorders: Advancing Translational and Clinical Applications

**DOI:** 10.7759/cureus.108691

**Published:** 2026-05-11

**Authors:** Abhay Singh, Rishikesh Kumar, Pradip Chauhan, Mallu Mallikarjuna, Ravi Thaker, Kumar Sambhav

**Affiliations:** 1 Department of Transfusion Medicine, Uttar Pradesh University of Medical Sciences, Saifai, IND; 2 Department of Microbiology, Indira Gandhi Institute of Medical Science, Patna, IND; 3 Department of Anatomy, All India Institute of Medical Sciences, Rajkot, Rajkot, IND; 4 Department of Virology, Sri Venkateswara University, Tirupati, IND; 5 Department of Physiology, Dr. N.D. Desai Faculty of Medical Science and Research, Dharmsinh Desai University, Nadiad, IND; 6 Department of Anatomy, All India Institute of Medical Sciences, Bilaspur, Bilaspur, IND

**Keywords:** crispr-cas9, gene editing, hematological disorders, precision medicine, translational applications

## Abstract

Clustered regularly interspaced short palindromic repeats-associated protein 9 (CRISPR-Cas9)-mediated gene editing has emerged as a pivotal advancement in hematological medicine, addressing genetic and regulatory abnormalities underlying inherited and acquired blood disorders. Conventional therapeutic strategies offer symptomatic control or partial disease modification, leaving substantial unmet clinical need for durable, mechanism-driven interventions. This narrative review aims to synthesize the translational and clinical progress of CRISPR-Cas9 applications in hematological disorders, with an emphasis on therapeutic strategies, safety considerations, and integration into clinical practice. A comprehensive literature evaluation was conducted using major biomedical databases, selecting peer-reviewed studies published between 2015 and 2025 that reported mechanistic, translational, or clinical relevance in hematology. Eligible literature included experimental investigations, clinical trials, and high-quality reviews, with exclusion of non-translational and germline-focused studies. This review highlights advances in hematopoietic stem cell editing, correction of hemoglobinopathies, immune cell engineering, and chimeric antigen receptor-based therapies, as well as progress in delivery platforms and high-fidelity nuclease designs. Key challenges related to off-target effects, genomic stability, manufacturing scalability, regulatory oversight, and ethical governance are critically evaluated. Emerging clinical trial outcomes demonstrate sustained therapeutic benefit and expanding feasibility across diverse hematological indications. Long-term clinical monitoring remains central to evaluating durability and safety. Collectively, current evidence supports CRISPR-Cas9 as a cornerstone technology in precision hematology, with continued optimization required to enable safe, equitable, and widespread clinical adoption.

## Introduction and background

Hematological disorders constitute a heterogeneous category of inherited and acquired diseases of blood cells involving their formation, functioning, and maintenance, and they are a significant complication of the worldwide morbidity, mortality, and health care costs [1]. The clinical burdens of sickle cell disease, thalassemia syndromes, aplastic anemia, myelodysplastic disorders, and hematological malignancies have chronic clinical presentations that are marked by frequent hospitalization, low quality of life, and permanent therapeutic addiction [2]. Advances in molecular hematology have clarified the genetic mutations, transcriptional dysregulation, and epigenetic changes underlying these conditions, enabling diagnostic classification and risk stratification [3]. Although these have been made, chronic molecular correction remains limited across various high-prevalence hematological disorders [4]. The existing treatment regimens still depend on pharmacological treatment, transfusion-based therapy, and hematopoietic stem cell transplantation [5]. Drug therapies tend to control disease expression rather than the underlying genetic defects [6]. Long-term treatment regimens bring about cumulative risks such as an iron overload, immune sensitization, and progressive organ damage [7]. Allogeneic stem cell transplantation is curative in certain disorders, but its clinical applicability is limited by donor supply, transplant-related toxicities, graft-versus-host disease, and immune-mediated complications [8]. In response to these clinical constraints, therapeutic approaches capable of delivering accurate and sustained, patient-specific molecular repair are required [9].

With the introduction of genome-editing technologies, therapeutic development in modern medicine has been redefined, as it is now possible to selectively alter disease-related genetic factors [5]. Clustered regularly interspaced short palindromic repeats-associated protein 9 (CRISPR-Cas9) is a highly promising platform that has emerged as the most popular platform because of its programmability, operational efficiency, and ability to work across various cellular environments [10]. The system allows cleavage of site-specific DNA under control of site-specific RNA sequences and enables disruption, correction, or transcriptional regulation of genes via endogenous DNA repair pathways [3]. The technical simplicity of editing compared to previous translation tools has accelerated the development of translation into a cornerstone of precision medicine in hematology, making CRISPR-Cas9 a cornerstone of this field [11]. Hematopoietic stem and progenitor cells are the best targets for gene-editing-based manipulations, as they have self-renewal capacity and can restore multilineage hematopoiesis [8]. Ex vivo editing therapies, followed by autologous reinfusion, have shown long-term therapeutic value in hemoglobinopathies by repairing pathogenic variants or reinstating fetal hemoglobin splicing [2]. Simultaneously occurring advances in the immune cell field have broadened the clinical treatment options for hematological malignancies, with CRISPR-enabled redesign of T lymphocytes and natural killer cells improving their antitumor selectivity, survival, and therapeutic intensity [12]. The developments described show the growing clinical applicability of gene-editing platforms in malignant and non-malignant hematological disorders [13].

Although the rate of clinical translation is increasing, critical scientific and implementation gaps remain. Long-term genomic integrity, off-target genomic alterations, and unexpected biological effects have remained key considerations in clinical risk assessment [7]. The challenges to widespread clinical implementation remain in delivery efficiency, editing accuracy in the quiescent stem cell population, and scalable manufacturing processes [3]. A further complication stems from ethical oversight standards, regulatory diversity, and cost-based limitations that affect equal access to gene-editing treatments [11]. The lack of an adequate alignment between molecular outcomes and real-world clinical endpoints also limits consistent uptake in routine hematology practice [5]. Additionally, regulatory governance of genome-editing therapies remains complex due to varying international regulatory frameworks, stringent safety validation requirements, and evolving policies governing advanced therapy medicinal products, which collectively influence clinical trial approval, manufacturing standards, and long-term monitoring obligations for gene-edited cellular therapies [4,8].

CRISPR-Cas9 gene editing is a rapidly evolving field at the intersection of molecular genetics, translational hematology, and personalized medicine. CRISPR, together with the Cas9 nuclease, functions as a programmable molecular system that allows researchers to identify and modify specific DNA sequences within the genome, thereby enabling targeted investigation or correction of disease-associated genetic alterations. Understanding the mechanistic basis, therapeutic strategies, safety considerations, and translational barriers of this technology is essential for responsible clinical development and application. This integration supports the progression of precision hematology and the development of mechanism-based interventions for diverse hematological disorders. This article presents a narrative review of CRISPR-Cas9-mediated genome editing in hematological diseases, summarising advances in molecular mechanisms, therapeutic applications in hemoglobinopathies and hematological malignancies, immune cell engineering including CAR-based approaches, delivery strategies, and safety considerations relevant to clinical translation. The review also highlights translational challenges, ethical and regulatory considerations, and areas requiring further mechanistic and clinical investigation before broader therapeutic implementation.

## Review

Methodology

Literature Search Strategy

An extensive literature search was conducted in PubMed, Scopus, Web of Science, and Google Scholar to locate peer-reviewed articles discussing CRISPR-Cas9-based gene editing in hematological diseases. Keywords consisted of CRISPR-Cas9, genome editing, hematopoietic stem cells, hematological malignancies, and clinical translation. Only eligible publications that were published between January 2015 and December 2025 were included in the search, reflecting the most important developments in the field of translational and clinical development. Relevant articles were also filtered by using reference lists to capture all articles. The search strategy aimed to capture mechanistic, translational, and clinical studies relevant to CRISPR-Cas9 applications in hematological disorders, ensuring comprehensive coverage of recent scientific developments within the defined timeframe.

Study Selection Criteria

Studies included were of original experimental research, clinical trials, and high-quality reviews that include mechanistic understanding, therapeutic use, or safety in hematological disorders. The exclusion criteria included studies lacking translational relevance, articles on non-hematological diseases, editorials, conference papers, and articles not in English. Titles and abstracts of retrieved records were evaluated for thematic relevance, followed by a full-text assessment to determine suitability for inclusion based on predefined criteria for clinical relevance, methodological rigor, and contribution to translational hematology.

Narrative Review Approach and Rationale

This study was conducted as a narrative review rather than a systematic review; therefore, strict PRISMA-based screening procedures and flow diagram reporting were not applied. Narrative reviews aim to synthesize and critically interpret existing evidence to provide conceptual understanding and thematic integration of emerging scientific developments, rather than to aggregate quantitative evidence. No meta-analysis or statistical synthesis of data was performed, as the objective of the review was qualitative interpretation and conceptual integration of published evidence rather than quantitative comparison of study outcomes. Accordingly, the literature selection process prioritized relevance to mechanistic insights, translational progress, and clinical applicability of CRISPR-Cas9 technologies in hematological disorders.

Data Evaluation and Synthesis

The qualitative evaluation of the selected literature was conducted based on the level of scientific rigor and clinical applicability to inform the synthesis of findings relevant to present practice in hematology. Selected studies were interpreted through thematic synthesis focusing on molecular mechanisms, therapeutic strategies, clinical translation, safety considerations, and regulatory challenges associated with CRISPR-Cas9-mediated gene editing in hematological diseases. This approach enabled integration of diverse forms of evidence to provide a comprehensive overview of the evolving landscape of genome-editing applications in hematological medicine.

Molecular basis of CRISPR-Cas9 gene editing

CRISPR-Cas9 gene editing can be traced to an adaptive immune response observed in prokaryotes and has been modified for specific control of genome sequences in eukaryotic cells [14]. This system has been based on a single-guide RNA, which guides the endonuclease Cas9 to a complementary DNA sequence next to a protospacer adjacent motif (PAM), a short conserved DNA sequence located immediately downstream of the target site that is required for Cas9 binding and activation of DNA cleavage, enabling Cas9 to induce a site-specific double-strand break (DSB) in the target DNA [15]. The Watson-Crick base pairing between the guide RNA and the target locus ensures target recognition accuracy and provides a programmable platform for repairing pathogenic variants in hematological diseases [4].

After DNA is cleaved, the repair mechanisms in cells dictate the final outcome of the genome. Non-homologous end joining is a fast repair mechanism that adds or removes small insertions or deletions to break down a specific gene or alter its regulation [10]. Homology-directed repair allows faithful sequence fix or gene insertion in situations where an exogenous donor template is available and has been utilized to repair monogenic blood diseases [16]. A balance between these repair pathways determines both their ability to repair and their therapeutic reliability, especially in hematopoietic stem and progenitor cells, where cell-cycle status dictates the choice of repair pathway [6]. In addition to these repair mechanisms, Cas9-induced DSB can activate cellular DNA damage response pathways, particularly the p53-mediated stress response, which may lead to transient cell-cycle arrest or apoptosis in edited cells and may influence editing efficiency and engraftment potential in hematopoietic stem and progenitor cells [7]. In long-lived stem cell populations, sustained activation of such pathways may theoretically create selective pressure favoring p53-deficient clones, raising concerns regarding long-term genomic stability and oncogenic risk following therapeutic genome editing [12].

Emerging evidence also indicates that genome repair outcomes following CRISPR-Cas9 cleavage are not limited to small insertions or deletions generated by non-homologous end joining. Larger genomic alterations, including extended deletions, chromosomal inversions, and translocations, may arise during DSB repair, particularly in proliferative hematopoietic cell populations, where structural genomic alterations are associated with leukemogenesis [3]. These findings highlight the importance of comprehensive genomic characterization when CRISPR-based editing strategies are applied in therapeutic hematology [9]. Off-target editing remains an important consideration, particularly in long-lived hematopoietic stem cells, where even rare unintended genomic alterations may persist and clonally expand over time. Mutations affecting oncogenes, tumor suppressor genes, or regulatory genomic regions may therefore carry long-term biological consequences, necessitating highly stringent thresholds for editing specificity and extensive preclinical evaluation using genome-wide off-target detection methods [5].

Furthermore, the possibility of clonal expansion following genome editing must be considered in hematological applications. Selective growth advantages arising from editing-induced genomic alterations, modulation of the DNA damage response pathway, or structural genomic rearrangements may result in clonal dominance within the edited stem cell pool. Consequently, long-term post-transplantation monitoring, genomic surveillance, and careful assessment of clonal dynamics are critical components of clinical translation of CRISPR-Cas9 therapies in hematological disorders [11].

The CRISPR-Cas9 engineering has improved accuracy and flexibility in hematological systems. High-fidelity versions of Cas9 minimize off-target cleavage without compromising on-target performance, thereby enhancing genomic stability during therapeutic editing [17]. Guide RNA engineering strategies have also been developed to improve targeting specificity, including truncated guide RNAs, chemically modified single-guide RNAs, rational computational design algorithms for target sequence optimization, and scaffold modifications that enhance Cas9-sgRNA binding stability and target discrimination [12]. These approaches reduce unintended genome interactions and improve editing precision in complex hematopoietic genomes [9]. Such improvements enable safer manipulation of stem cells and immune effector cells, as well as their use in autologous transplantation or immunotherapy [7]. CRISPR-Cas9 has growing translational applicability due to its adaptability across a wide variety of hematopoietic cell types. Ex vivo editing of stem cells enables direct manipulation under improved conditions and the reinfusion of the stem cells to achieve permanent hematopoietic reconstitution [12]. Hematopoietic stem and progenitor cells represent particularly important targets for genome editing because of their long-term self-renewal capacity, ability to undergo clonal expansion, and sensitivity to genotoxic stress, which together influence both the durability and safety of therapeutic genome modifications [8]. In immune cell engineering, multiplex gene editing enables simultaneous modulation of immune checkpoints, antigen receptors, and signaling pathways, improving functional persistence and antitumor effects [5]. The result of such mechanistic versatility positions CRISPR-Cas9 as the basis for precision hematological interventions. Table 1 shows the key molecular components, DNA repair pathways, and hematological relevance underlying CRISPR-Cas9-mediated gene editing.

**Table 1:** Molecular components and repair pathways of CRISPR-Cas9 in hematology CRISPR: clustered regularly interspaced short palindromic repeats, Cas9: CRISPR-associated protein 9, sgRNA: single-guide RNA, DNA: deoxyribonucleic acid, NHEJ: non-homologous end joining, HDR: homology-directed repair, PAM: protospacer adjacent motif

Component	Functional role	Mechanistic outcome	Relevance to hematology	Key references
sgRNA	Target sequence recognition	Guides Cas9 to a specific genomic locus	Enables disease-specific targeting	[17]
Cas9	Endonuclease activity	Induces double-strand DNA breaks	Initiates genomic modification	[14]
NHEJ	Error-prone repair pathway	Gene disruption or regulatory alteration	Useful in oncogene inactivation	[2]
HDR	Template-driven repair	Precise sequence correction	Correction of monogenic mutations	[9]
PAM	Target site requirement	Ensures correct Cas9 binding	Enhances targeting specificity	[18]

Hematopoietic stem cells as therapeutic targets

Hematopoietic stem cells constitute a key therapeutic cellular platform for gene-editing-based interventions in hematological diseases because of their distinctive biological characteristics, which facilitate lifelong hematopoiesis, and have recently been demonstrated in clinical gene-editing trials targeting hemoglobinopathies [19]. These cells possess self-renewal capacity and multilineage differentiation potential, allowing the production of erythroid, myeloid, and lymphoid lineages after successful genetic modification [20]. However, the long-term persistence and therapeutic durability of edited hematopoietic stem cells depend on several biological determinants, including efficient engraftment following transplantation, preservation of stem cell fitness after genome editing, maintenance of self-renewal capacity, and the absence of clonal dominance within the edited stem cell population [7]. Stem cell-based correction of pathogenic variants can provide durable restoration of defective hematopoietic populations, reducing the need for repeated therapeutic interventions [15]. Congenital blood diseases such as sickle cell disease and thalassemia syndromes arise from well-characterized genetic defects in hematopoietic lineages, making hematopoietic stem cells an ideal substrate for targeted genomic correction [8]. Editing approaches aimed at restoring normal gene expression or modulating regulatory elements controlling fetal hemoglobin expression demonstrate significant therapeutic potential and have been validated in recent CRISPR-Cas9 clinical trials targeting the BCL11A erythroid enhancer [21].

Despite these advantages, several biological constraints influence the effectiveness of genome editing in hematopoietic stem cells. The population of true long-term repopulating HSCs within harvested stem cell pools is relatively limited, which may restrict the number of cells capable of sustaining durable hematopoiesis following editing and transplantation [6]. The quiescent state of many HSCs also influences editing efficiency, as homology-directed repair mechanisms are strongly dependent on cell-cycle progression and therefore occur less efficiently in non-dividing stem cells [10]. Additionally, Cas9-induced DNA DSB can activate cellular DNA damage response pathways, potentially affecting stem cell survival and functional integrity during ex vivo manipulation [12]. Prolonged ex vivo culture conditions required for editing and expansion may further compromise stem cell fitness, leading to partial loss of stemness or altered differentiation potential, representing an important translational challenge for therapeutic HSC editing strategies [9]. Gene-editing strategies targeting HSCs may therefore enable reprogramming of aberrant hematopoietic pathways in both inherited disorders and acquired conditions, such as marrow failure syndromes and clonal hematopoiesis [13].

**Figure 1:**
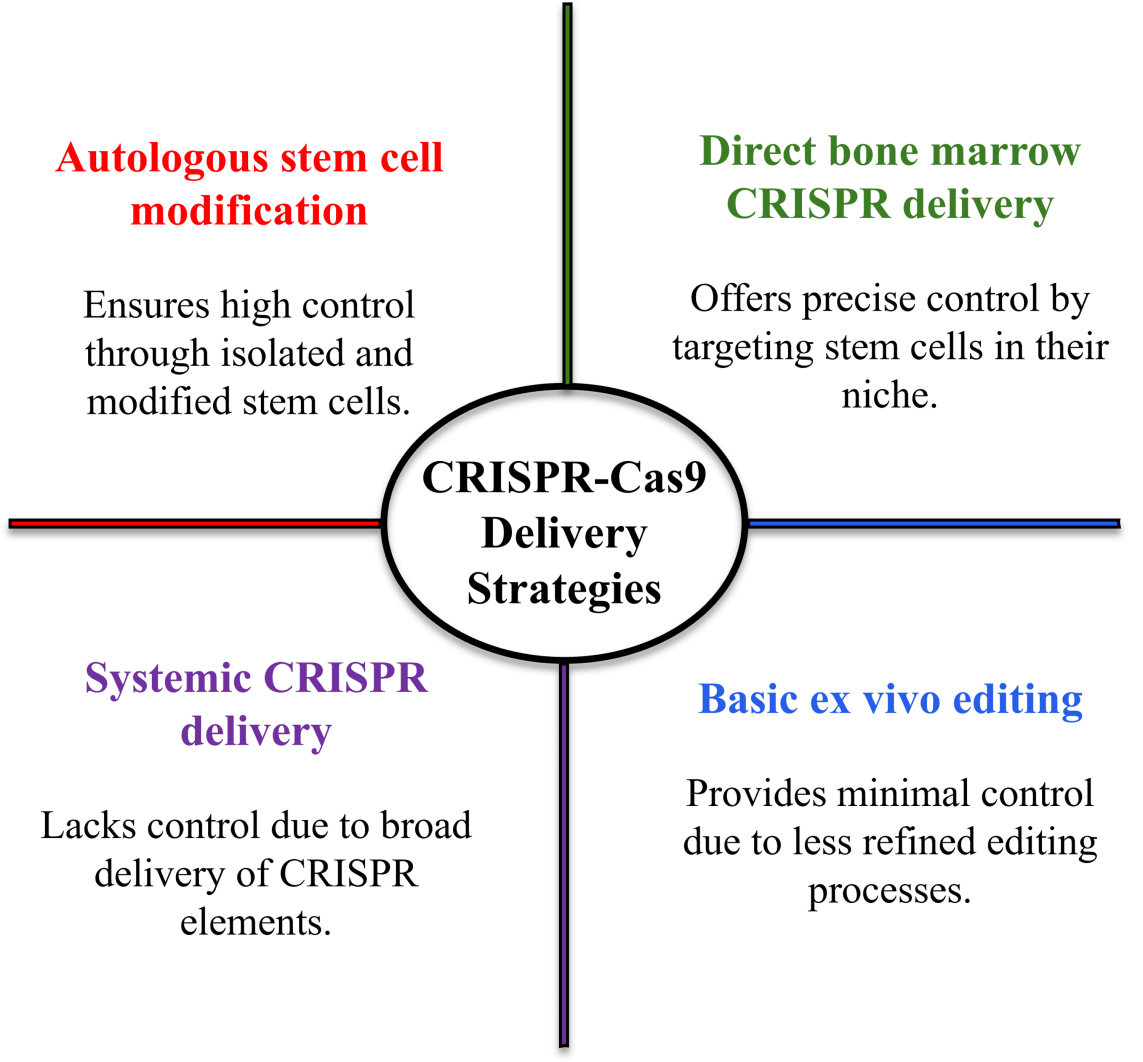
CRISPR-Cas9 delivery strategies in hematological therapy CRISPR: clustered regularly interspaced short palindromic repeats, Cas9: CRISPR-associated protein 9 Image Credit: Authors using PowerPoint (Microsoft Corp., Redmond, WA, USA)

Current clinical translation primarily relies on ex vivo editing approaches, which involve isolation of autologous hematopoietic stem cells, controlled CRISPR-Cas9-based modification, and reinfusion following conditioning regimens [19]. Ex vivo platforms enable stringent quality control, assessment of editing efficiency, and evaluation of off-target effects prior to clinical administration [11]. Recent clinical trials using CRISPR-edited autologous HSCs, including therapies such as exagamglogene autotemcel (exa-cel), have demonstrated durable induction of fetal hemoglobin and sustained clinical improvement in patients with sickle cell disease and transfusion-dependent β-thalassemia [20]. Emerging in vivo editing approaches aim to deliver CRISPR components directly to stem cells within the bone marrow niche, potentially simplifying therapeutic procedures [8]. However, challenges related to delivery specificity, editing efficiency, and systemic safety remain under active investigation. Ongoing optimization of delivery vectors and engineered Cas variants continues to improve the feasibility of clinically applicable in vivo stem cell editing platforms. Figure 1 shows major CRISPR-Cas9 delivery approaches based on control, precision, and clinical applicability in hematological gene editing.

CRISPR-Cas9 applications in hemoglobinopathies

Hemoglobinopathies are a significant category of inherited blood diseases, which are progressive anemias caused by structural changes or quantitative deficiencies in globin chain synthesis, resulting in systemic complications [22]. The most common types are sickle cell disease and β-thalassemia. Sickle cell disease arises from a single point mutation in the β-globin gene that alters hemoglobin structure. In contrast, β-thalassemia is genetically heterogeneous and results from more than 300 identified mutations affecting the β-globin gene, including promoter mutations, splice-site alterations, nonsense mutations, and gene deletions that impair β-globin synthesis [17]. Recent breakthroughs in molecular genetics have made CRISPR-Cas9 an excellent tool for treating disorders with a genetic basis with high precision via genome editing [10]. CRISPR-Cas9 approaches aimed at correcting the pathogenic point mutation in the β-globin gene or regulating regulatory elements that affect hemoglobin composition are the primary approaches in sickle cell disease [23]. The goal of direct gene correction is to restore normal hemoglobin production, reduce erythrocyte sickling, and prevent vaso-occlusive crises resulting from sickling [14]. Other methods aim to disrupt transcriptional repressors of fetal hemoglobin, thereby enabling long-term expression of γ-globin chains to replace defective adult hemoglobin [24]. High levels of fetal hemoglobin confer disease-protective effects, which is why this strategy can be considered a clinically viable intervention [18].

Despite these therapeutic advances, several translational challenges remain in CRISPR-Cas9-based correction of hemoglobinopathies. Potential off-target genome-editing events and DSB-associated genotoxicity are important safety considerations, particularly in long-lived hematopoietic stem and progenitor cells [12]. Additionally, the efficiency of homology-directed repair can vary considerably in HSPCs due to cell-cycle dependence and the quiescent nature of many stem cell populations, which may limit the precision of direct gene correction strategies [7]. Furthermore, regulatory oversight and long-term clinical monitoring remain essential, as gene-edited stem cell therapies require careful evaluation of genomic stability, durability of therapeutic benefit, and potential delayed adverse events following transplantation [11].

In β-thalassemia, CRISPR-Cas9-based strategies aim to restore balanced globin chain synthesis by correcting β-globin gene mutations and by precisely modulating globin gene switch regulators, including activators and repressors [13]. In current clinical translation, a major therapeutic strategy involves targeted disruption of the erythroid-specific enhancer of the BCL11A gene, a key transcriptional repressor of fetal hemoglobin expression, thereby enabling selective reactivation of γ-globin production in erythroid cells [10]. This lineage-restricted targeting approach is designed to increase fetal hemoglobin levels while preserving BCL11A function in other hematopoietic lineages, particularly lymphoid cells, where the gene plays an essential role in normal immune development [22]. Reactivation of fetal hemoglobin by destabilizing silencing elements has become a common therapeutic approach across hemoglobinopathies and has provided mutation-independent clinical benefit [10]. These therapies reduce unproductive erythropoiesis and transfusion dependence, targeting fundamental pathologic processes [22]. Ex vivo editing of autologous hematopoietic stem cells is the most common translational pathway in hemoglobinopathy treatment. Conditioned, modified stem cells are reinfused, enabling long-term hematopoietic reconstitution with therapeutically optimized hemoglobin expression [25]. According to clinical reports, CRISPR-based interventions result in permanent engraftment, prolonged induction of fetal hemoglobin, and improved hematological values [19].

Despite encouraging clinical outcomes, careful evaluation of genomic safety remains essential for therapeutic implementation. Comprehensive off-target assessment strategies are required to detect unintended genomic edits, particularly in long-lived hematopoietic stem cell populations where rare events may persist following transplantation [11]. In addition to small insertions or deletions, CRISPR-induced DSB may occasionally generate structural genomic alterations such as larger deletions or chromosomal rearrangements, which necessitate detailed genomic characterization during preclinical and clinical evaluation [7]. Furthermore, monitoring of clonal expansion within edited stem cell populations is critical, as selective growth advantages could theoretically lead to clonal dominance after transplantation [12]. Consequently, long-term clinical surveillance and genomic monitoring remain integral components of CRISPR-based therapeutic strategies in hemoglobinopathies to ensure sustained safety and therapeutic durability [5]. The main molecular targets, interventional strategies, and translational results of CRISPR-Cas9 interventions in hemoglobinopathies are shown in Table 2.

**Table 2:** CRISPR-Cas9 strategies in hemoglobinopathies CRISPR: clustered regularly interspaced short palindromic repeats, Cas9: CRISPR-associated protein 9, SCD: sickle cell disease, HbF: fetal hemoglobin, DNA: deoxyribonucleic acid, BCL11A: B-cell lymphoma/leukemia 11A

Target disorder	Editing strategy	Molecular target	Therapeutic outcome	Clinical status
SCD	Gene correction	β-globin mutation	Reduced erythrocyte sickling	[22]
SCD	HbF induction	BCL11A enhancer	Increased fetal hemoglobin	[19]
β-Thalassemia	HbF reactivation	γ-globin repression sites	Improved globin balance	[24]
β-Thalassemia	Gene repair	β-globin gene	Reduced transfusion burden	[20]
Combined	Regulatory editing	Globin locus control regions	Sustained hemoglobin normalization	[16]

Gene editing strategies in thalassemia syndromes

Thalassemia syndromes are inherited disorders caused by defects in globin chain synthesis, leading to an imbalance in hemoglobin composition, ineffective erythropoiesis, and chronic anemia [26]. Two principal molecular forms are recognized: α-thalassemia, which results from deletions or mutations affecting the α-globin genes (HBA1 and HBA2) on chromosome 16, and β-thalassemia, which arises from mutations in the β-globin gene on chromosome 11 that reduce or abolish β-globin synthesis [22]. In β-thalassemia, the deficiency of β-globin chains leads to accumulation and precipitation of excess α-globin chains within erythroid precursors, generating oxidative stress, membrane damage, and apoptosis of developing erythroblasts, thereby contributing to ineffective erythropoiesis and chronic anemia [10]. Clinically, thalassemia syndromes are commonly classified into thalassemia major, thalassemia intermedia, and thalassemia minor based on the severity of globin imbalance, transfusion requirements, and clinical manifestations [19].

Traditional management techniques, such as lifelong transfusion support and iron chelation therapy, help to alleviate complications caused by the disease but do not help to overcome the genetic etiology [22]. Iron overload in thalassemia arises not only from repeated transfusions but also from increased intestinal iron absorption driven by suppression of the iron-regulatory hormone hepcidin, which contributes independently to systemic iron toxicity and organ damage [27]. Recent developments in molecular therapeutics have positioned CRISPR-Cas9-based gene editing as a highly promising modality for achieving sustained genomic remediation [10]. Thalassemia gene-editing approaches mostly focus on control mechanisms of globin gene expression. Contemporary strategies include targeted disruption of the erythroid enhancer of the BCL11A gene to reactivate fetal hemoglobin expression, as well as direct correction of pathogenic mutations in the β-globin locus using precision genome-editing technologies [19]. Interference with transcriptional repressors of γ-globin, including BCL11A and other regulators involved in hemoglobin switching, has been developed as a largely mutation-independent strategy, as fetal hemoglobin induction can provide clinical benefit across diverse β-thalassemia genotypes, regardless of the underlying β-globin mutation [19]. In translational practice, editing is frequently directed toward erythroid-specific regulatory elements (such as the BCL11A erythroid enhancer) rather than global disruption of coding regions, supporting lineage-restricted fetal hemoglobin reactivation while minimizing broader hematopoietic effects [10]. High levels of fetal hemoglobin shift the globin chain equilibrium and enhance erythrocyte survival, thereby reducing anemia severity across thalassemia genotypes [27]. Creating a specific change in enhancer elements within the globin locus further controls hemoglobin switching and is useful for therapeutic maintenance of expression.

Direct correction of pathogenic variants in the β-globin gene is another approach, particularly for a specific thalassemia mutation [23]. Accurate editing restores normal β-globin synthesis, thereby reducing excess δ-globin production and associated cellular toxicity [20]. These approaches emphasize precision and preservation of genomic integrity, meeting key safety criteria for clinical translation [17]. Editing autologous hematopoietic stem cells is the prevailing translational approach for managing thalassemia. After myeloablative conditioning, ex vivo CRISPR-Cas9 modification of stem cells is performed under controlled conditions, and the cells are reinfused afterward [5]. By using this method, long-term hematopoietic reconstitution with genetically repaired progenitor cells can be achieved with minimal immunological complications associated with allogeneic reconstitution [28]. Edited stem cell therapy has been shown to result in stable engraftment, persistent improvement in hemoglobin levels, and reduced transfusion dependence. The continuous streamlining of delivery effectiveness and editing fidelity further facilitates the incorporation of gene-editing plans into sophisticated thalassemia treatment pathways.

CRISPR-Cas9 in hematological malignancies

Hematological malignancies are diseases driven by genetic and epigenetic alterations that impair normal homeostasis of hematopoiesis, survival signals, and immune controls, resulting in unregulated growth of malignant clones [29]. Recurrent oncogenic mutations, chromosomal rearrangements, and dysregulated transcriptional programs are observed in leukemias, lymphomas, and multiple myeloma and contribute to disease progression and therapy resistance [8]. Traditional treatment modalities, such as chemotherapy, targeted agents, and stem cell transplantation, have variable clinical outcomes and are still limited by toxicity, relapse, and clonal evolution [22]. CRISPR-Cas9 gene editing has emerged as a valuable experimental platform for investigating oncogenic drivers and exploring potential therapeutic strategies in malignant hematopoietic cells; however, its clinical translation remains constrained by several challenges, including efficient in vivo delivery, potential off-target editing events, DSB-associated genotoxicity, chromosomal rearrangements, and the possibility of p53-mediated cellular selection following genome editing [27]. Functional suppression of tumor growth and survival can be achieved by targeted disruption of gain-of-function mutations, fusion oncogenes, and aberrantly activated signaling pathways [30]. Edits targeting tumor suppressor pathways enable mechanistic interpretation of malignant transformation and the identification of vulnerabilities that can be exploited to develop therapeutic strategies [20]. Multiplex editing also enables simultaneous targeting of cooperative oncogenic networks to address the genetic heterogeneity of hematological cancers [31].

**Figure 2:**
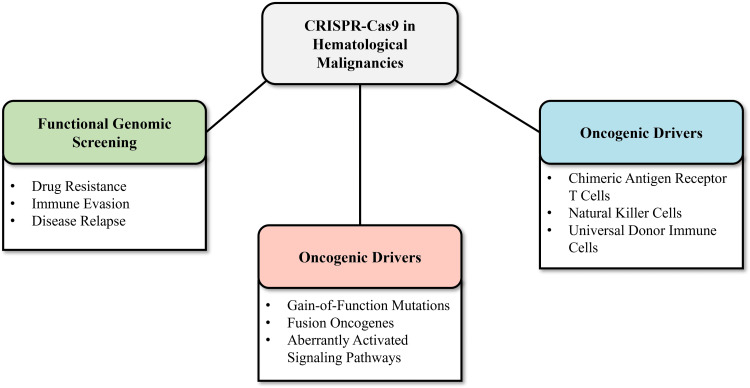
Applications of CRISPR-Cas9 in hematological malignancies CRISPR: clustered regularly interspaced short palindromic repeats, Cas9: CRISPR-associated protein 9 Image Credit: Authors using PowerPoint (Microsoft Corp., Redmond, WA, USA)

CRISPR-Cas9-based immune applications have broadened therapeutic opportunities for treating leukemia and lymphoma. With gene editing, it is possible to improve chimeric antigen receptor T-cells (CAR-T) and natural killer cells by deleting inhibitory receptors, modulating exhaustion-related mechanisms, and optimizing antigen recognition [18]. The modifications enhance cytotoxic persistence, antitumor activity, and resistance to the immunosuppressive tumor microenvironment [26]. Specific elimination of endogenous T-cell receptors or human leukocyte antigen complex components promotes the production of universal-donor immune cells, advancing strategies for allogeneic immunotherapy [32]. Outside the therapeutic engineering field, CRISPR-Cas9 is being used to support functional genomic screening of the genes linked to drug resistance, immune evasion, and disease relapse [15]. The information obtained from such methods informs the rational combination of therapies and precision-targeted interventions [30]. Further improvements in delivery platforms, safety measures, and genomic fidelity enhance the translational applicability of CRISPR-Cas9 in hematological malignancies, making it an important component of more efficient oncologic care systems. Figure 2 shows major CRISPR-Cas9 applications in hematological malignancies, encompassing functional genomic screening, oncogenic driver targeting, and immune-based therapies.

Immuno-hematology and CAR-based gene editing

The use of CRISPR-Cas9-mediated gene editing in immuno-hematology has expanded significantly, particularly in enhancing the design and functional optimization of CAR-based cellular therapies rather than contributing to the initial development of CAR-T technology, which was originally established using viral transduction approaches [25]. CAR-T and CAR-NK cell systems enable selective targeting and elimination of malignant hematopoietic cells via engineered antigen specificity, offering important therapeutic options for refractory leukemias and lymphomas [14]. Structurally, CAR constructs typically consist of an extracellular antigen-recognition domain derived from a single-chain variable fragment, a transmembrane domain, and intracellular signaling modules including CD3ζ activation domains together with co-stimulatory domains such as CD28 or 4-1BB that enhance T-cell activation, proliferation, and persistence [33]. These engineered receptors are commonly directed against tumor-associated antigens such as CD19 in B-cell malignancies or B-cell maturation antigen in multiple myeloma, enabling selective recognition of malignant cells [19]. Upon antigen engagement, CAR-modified immune cells exert cytotoxic activity through mechanisms including perforin-granzyme release, cytokine secretion, and activation of apoptotic signaling pathways within tumor cells [28].

Immune exhaustion, antigen escape, graft rejection, and off-target cytotoxicity remain major challenges affecting long-term therapeutic durability [33]. CRISPR-Cas9 genome editing has therefore been explored to optimize CAR-based therapies by enabling precise genetic modifications that enhance cellular persistence, safety, and therapeutic efficacy [10]. Specific elimination of suppressive immune checkpoint receptors can enhance immune cell survival and antitumor activity within the immunosuppressive tumor microenvironment [19]. Targeted editing of transcriptional regulators associated with T-cell exhaustion may also help sustain cytotoxic function during prolonged antigen exposure [28]. Simultaneously, endogenous T-cell receptor expression can be disrupted to reduce the risk of graft-versus-host disease and to facilitate the development of universal-donor CAR-T platforms suitable for allogeneic therapeutic applications [10].

CRISPR-mediated genome editing has also been applied to improve CAR-NK cell engineering through targeted gene knockouts or knock-ins that regulate natural killer cell activation and persistence [29]. For instance, disruption of inhibitory signaling regulators such as cytokine-inducible SH2-containing protein or programmed cell death protein-1, together with modulation of killer-cell immunoglobulin-like receptors, can enhance tumor recognition and cytotoxic effector function in engineered NK cells [29]. These genetic modifications improve NK-cell activation by lowering inhibitory signaling thresholds, thereby increasing perforin- and granzyme-mediated tumor killing and cytokine release against malignant targets [33]. Edited CAR-NK cells have demonstrated favorable safety profiles, including lower incidence of cytokine release toxicity and broader applicability across diverse patient populations [33]. These characteristics position CAR-NK therapies as complementary immunotherapeutic modalities in the treatment of hematological malignancies. Precision genome editing can also improve therapeutic specificity by refining antigen-binding domains and intracellular signaling modules, thereby enhancing discrimination between malignant and normal hematopoietic cells [20]. Multiplex genome-editing strategies further allow simultaneous modulation of immune activation pathways and apoptotic regulators, improving therapeutic selectivity and clinical safety [34]. The main CRISPR-Cas9-mediated changes, molecular targets, and clinical outcomes of CAR immunotherapies in hematological malignancies are summarized in Table 3.

**Table 3:** CRISPR-Cas9 CAR-based immunotherapies in hematological malignancies CAR: chimeric antigen receptor, CAR-T: chimeric antigen receptor T-cell, CAR-NK: chimeric antigen receptor natural killer cell, CRISPR: clustered regularly interspaced short palindromic repeats, Cas9: CRISPR-associated protein 9, PD-1: programmed cell death protein 1, TCR: T-cell receptor, TRAC: T-cell receptor alpha constant, KIR: killer-cell immunoglobulin-like receptor, HLA: human leukocyte antigen

Cell type	CRISPR editing strategy	Molecular target	Functional outcome	Representative clinical trial (phase)	In-text citation
CAR-T	Immune checkpoint deletion	PD-1 gene	Enhanced cellular persistence	NCT04035434 (Phase I)	[30]
CAR-T	TCR knockout	TRAC locus	Reduced graft rejection	NCT03399448 (Phase I)	[27]
CAR-NK	Receptor modulation	KIR genes	Increased cytotoxicity	NCT04245722 (Phase I)	[23]
CAR-NK	Signaling enhancement	Cytokine receptors	Improved survival and function	NCT03940820 (Phase I)	[34]
Universal CAR	Multiplex genome editing	HLA components	Off-the-shelf therapy feasibility	NCT03166878 (Phase I)	[32]

Delivery platforms for CRISPR systems

The effective and safe transfer of CRISPR-Cas9 elements remains a significant factor in therapeutic success in hematological studies [35]. The use of delivery platforms affects editing efficiency, cell specificity, genomic integrity, and overall clinical feasibility, particularly in hematopoietic stem cells and immune effector populations [26]. Both non-viral and viral delivery systems have been investigated to address the various biological and translational needs in hematological disorders [36]. Representative viral delivery systems include lentiviral vectors and adeno-associated viral vectors, which are widely used to introduce gene-editing components or donor templates in experimental and clinical settings [30]. Lentiviral and adeno-associated viral systems, which are examples of viral vectors, are highly efficient at transduction and can stably express gene-editing components [30]. Lentiviral vectors facilitate transfer to both dividing and non-dividing hematopoietic cells, thereby enabling permanent alterations in ex vivo stem cell editing protocols [25]. Viral vectors based on adeno-associated viruses exhibit lower immunogenicity and transient expression profiles and are used when it is necessary to control Cas9 activity with high temporal accuracy [37]. Vectors' genotoxicity and size-constrained integration are also important factors affecting vector choice in clinical settings [20].

In addition to viral approaches, several non-viral delivery strategies have been explored, including electroporation of Cas9-guide RNA ribonucleoprotein complexes, lipid nanoparticle systems, and polymer-based nanoparticle carriers that enable transient and controlled delivery of genome-editing components [36]. Such non-viral platforms are particularly relevant for hematopoietic stem cell editing because transient exposure to CRISPR components may reduce prolonged nuclease activity and limit unintended genomic alterations [35]. In therapeutic applications targeting hematopoietic stem cells, delivery strategies must also prioritize preserving stem cell fitness and self-renewal capacity, minimize activation of the DNA damage response, and avoid chromosomal rearrangements that could compromise genomic stability or long-term engraftment following transplantation [26].

Non-viral delivery systems have gained prominence owing to their good biosafety profiles and lower risk of insertional mutagenesis [31]. This is achieved by delivering ribonucleoprotein complexes via electroporation to rapidly introduce the Cas9 protein and guide RNA into cells, enabling transient editing with reduced off-target effects [6]. Lipid nanoparticles offer a scalable and reproducible delivery platform with flexibility for both ex vivo and emerging in vivo applications [13]. Physical and chemical delivery routes demonstrate enhanced regulatory acceptability due to predictable pharmacokinetics and controlled exposure time [32]. The clinical suitability of delivery platforms is determined by balancing editing efficiency, safety, and manufacturability [11]. Transient delivery systems minimize prolonged nuclease exposure and promote genomic stability in edited cell populations [6]. Scalability for large-scale cell processing further dictates translational viability, particularly for autologous cell therapies [17]. Continued advances in vector engineering, editing specificity, and delivery accuracy are expanding the clinical application of CRISPR-Cas9-based interventions in hematological medicine.

Safety, off-target effects, and genomic stability

The translation of CRISPR-Cas9-mediated gene editing into the clinical setting of hematological diseases requires careful management of safety parameters, particularly off-target activity and long-term genomic stability [33]. Off-target cleavage may occur when guide RNA sequences partially match unintended genomic loci; however, editing specificity is also influenced by additional factors, including guide RNA sequence composition and GC content, chromatin accessibility at potential target sites, tolerance of the PAM, and the intracellular concentration and exposure duration of the Cas9-sgRNA complex [37]. These factors collectively influence binding affinity and cleavage probability, thereby affecting the likelihood of unintended genome modification [17]. Such risks are especially relevant in hematopoietic stem cells because of their long-term repopulating capacity, which may allow unintended genomic alterations to persist following transplantation [17]. In addition to small insertions or deletions, CRISPR-induced DSB may occasionally generate larger genomic alterations, such as extended deletions, chromosomal translocations, or complex structural rearrangements, that can compromise genomic integrity in long-lived stem cell populations [17]. Activation of cellular DNA damage response pathways, including p53-mediated stress signaling, may further influence clonal selection dynamics following genome editing [12].

Advances in nuclease engineering have led to the development of high-fidelity Cas9 variants designed to improve target discrimination and reduce unintended editing events [38]. Modified nucleases with enhanced DNA-binding specificity demonstrate markedly reduced off-target interactions while maintaining on-target editing efficiency [39]. Examples include engineered variants such as enhanced specificity Cas9 (eSpCas9) and high-fidelity Cas9 (HiFi Cas9), which limit non-specific DNA interactions through structural modifications of the nuclease domain [38]. Additional strategies aimed at improving editing precision include paired nickase systems and truncated guide RNA designs, which reduce the probability of unintended DSB by requiring higher-order DNA recognition [14]. More recently, alternative genome-editing platforms, such as base editing and prime editing, have been developed to enable targeted nucleotide modification without inducing DSB, thereby potentially reducing genotoxic risk in therapeutic applications [12]. Transient delivery of Cas9-guide RNA ribonucleoprotein complexes, together with computational off-target prediction tools, further helps limit nuclease exposure and improve the preclinical identification of potential off-target sites [37].

Comprehensive genomic surveillance strategies are also essential for evaluating long-term safety following gene-edited cell transplantation. Genome-wide off-target detection assays allow systematic identification of unintended edits during preclinical screening, while clonal tracking and extended post-transplantation follow-up support the evaluation of genomic stability and hematopoietic reconstitution [35]. Genomic stability, therefore, remains a critical determinant of therapeutic safety and durability. Strategies aimed at limiting large deletions, preserving chromosomal architecture, and maintaining balanced DNA repair responses contribute to the long-term integrity of edited genomes [38]. Integration of genome-editing strategies with cell-cycle regulation and optimized conditioning regimens may further enhance the stability of edited stem cell populations [40]. Continued optimization of nuclease design, delivery kinetics, and genomic monitoring frameworks is essential to align CRISPR-Cas9 technologies with clinical safety standards in hematological medicine.

Ethical, regulatory, and clinical trial landscape

The experimental development of CRISPR-Cas9-based gene editing in hematological conditions operates within a multilayered ethical and regulatory framework that prioritizes patient safety, genomic integrity, and responsible innovation [27]. Regulatory oversight of genome-editing therapies is conducted by national and international regulatory authorities such as the United States Food and Drug Administration and the European Medicines Agency, which require extensive preclinical evaluation, manufacturing quality control, and carefully designed clinical trials prior to therapeutic implementation [11]. Clinical studies involving gene-editing interventions are typically reviewed by institutional review boards and monitored by independent safety oversight mechanisms, such as data safety monitoring boards, to ensure ethical conduct, participant protection, and compliance with established regulatory standards [33]. Additionally, ex vivo gene-edited cellular products must be produced in good manufacturing practice-compliant environments that ensure product consistency, sterility, and traceability prior to clinical administration [30].

Somatic gene editing is generally regarded as ethically permissible in therapeutic contexts involving severe or life-limiting diseases; however, its acceptability remains conditional and is the subject of ongoing ethical discussion [11]. Ethical acceptability depends on several considerations, including careful evaluation of risk-benefit balance, robust informed consent procedures, equitable access to emerging therapies, and long-term clinical follow-up to monitor potential delayed or unforeseen adverse effects associated with permanent genomic modification [33]. Ethical governance in genome editing emphasizes informed consent, proportional risk-benefit assessment, and long-term follow-up to address uncertainties associated with permanent genomic modification [33]. Concerns surrounding unintended genetic alterations, avoidance of intergenerational effects, and equitable access remain central to ethical discussions in clinical hematology [30]. Additionally, the possibility of unknown long-term genomic risks continues to generate ethical debate regarding the responsible clinical implementation of gene-editing technologies [12]. The strict prohibition of germline genome modification in clinical practice reflects a strong international consensus aimed at preventing heritable genetic changes [12]. Transparency in trial design, outcome reporting, and long-term follow-up strengthens ethical accountability and public trust in gene-editing technologies [21].

Regulatory frameworks across regions are guided by common principles centered on safety validation, genomic stability assessment, manufacturing quality, and demonstration of clinical efficacy [33]. Regulatory authorities, therefore, require extensive preclinical evaluation of off-target effects, genomic stability, and functional consequences before approval for human use [37]. Advanced therapy classification pathways provide structured regulatory frameworks for evaluating gene-edited cellular products in accordance with evolving scientific and clinical standards [11]. Regulatory harmonization initiatives across jurisdictions facilitate efficient global development and the implementation of multinational clinical trials [30]. The international clinical trial landscape reflects accelerating translation of CRISPR-Cas9 therapies in hematology, particularly for hemoglobinopathies and refractory hematological malignancies [1]. Early-phase clinical trials primarily focus on safety, feasibility, and biological activity, whereas later-phase studies increasingly emphasize durability of response and clinical benefit [9]. Current trial designs are dominated by autologous ex vivo stem cell editing approaches supported by controlled manufacturing processes and predictable safety profiles. The continued expansion of clinical trial cohorts and geographic participation reflects increasing regulatory confidence and growing clinical maturity of CRISPR-Cas9-based therapeutic strategies.

Translational challenges and clinical integration

There are several structural and clinical challenges in translating CRISPR-Cas9-based therapeutics from experimental settings into routine hematological practice [33]. Scalability to manufacturing remains a major limitation, particularly for autologous cell-based interventions that require individualized cell collection, gene editing, expansion, and reinfusion for each patient [17]. These batch-based workflows demand highly specialized infrastructure, stringent quality control, and reproducible editing efficiency, resulting in prolonged production timelines and operational complexity [11]. Substantial cost burdens further constrain clinical integration. Humanized manufacturing processes, high-technology vector production, and extended hospitalization during conditioning regimens impose significant financial strain on healthcare systems [30]. Limited availability of centralized manufacturing facilities, especially in low- and middle-income regions, further restricts accessibility despite a high burden of hematological disease [37]. Economic sustainability and reimbursement frameworks often lag behind scientific progress, limiting adoption even when therapeutic benefit has been demonstrated [11].

Workforce-related barriers also affect clinical integration. Successful implementation requires a multidisciplinary clinical and technical workforce, including hematologists, transplant specialists, molecular biologists, and regulatory experts [21]. Insufficient exposure of clinicians to gene-editing technologies influences referral pathways and patient selection processes [29]. The absence of fully established clinical guidelines for eligibility assessment, adverse event monitoring, and long-term follow-up introduces variability in clinical implementation [33]. Regulatory and logistical complexities further hinder translational success. Divergent regulatory standards across jurisdictions complicate the execution of multinational trials and the approval processes [30]. Post-treatment surveillance obligations extend beyond conventional therapeutic models and require integration of long-term follow-up frameworks into standard care pathways [18]. Inadequate distribution of genomic surveillance systems and adverse event reporting infrastructure across healthcare settings limits comprehensive safety monitoring [37]. Despite these challenges, clinical integration is advancing through scalable manufacturing innovations, cost-reduction strategies, clinician training initiatives, and regulatory alignment efforts [11]. Development of off-the-shelf allogeneic platforms, automation of cell processing, and centralized manufacturing models are improving feasibility and accessibility. Strategic alignment of scientific innovation with healthcare delivery systems remains essential for widespread clinical implementation of CRISPR-Cas9-based therapies in hematological disease.

Limitations and future recommendations

The current synthesis is limited in several ways. It focuses on the published literature, which may introduce publication bias and lead to an overrepresentation of positive or inconclusive results. The heterogeneity in study designs, delivery platforms, and outcome measures precludes direct comparability and quantitative inference. A rapid pace of technological development in CRISPR-Cas9 systems poses the risk of the partial obsolescence of previous discoveries over the considered period. Limited long-term follow-up evidence hampers robust assessment of genomic integrity, persistence of clinical benefit, and delayed adverse events following gene-edited cellular therapies.

The future paths must focus on standardized reporting systems, balanced clinical endpoints, and longitudinal monitoring across translational programs. Generalizability and equity might be enhanced by including larger, more diverse populations and healthcare settings. Further development of high-fidelity nucleases, cell-targeted delivery methods, and scalable production pipelines should be given particular consideration. Possibly, the safe and effective implementation of gene-editing therapies in routine hematological practice can be further supported by integrating real-world evidence, health-economic evaluation, and structured clinician training initiatives.

## Conclusions

CRISPR-Cas9 gene editing has emerged as a transformative platform in hematological medicine, offering precise and programmable modification of disease-causing genetic and regulatory elements in both inherited and acquired blood disorders. Recent advances in molecular targeting, hematopoietic stem cell manipulation, immune cell engineering, and delivery technologies have generated strong translational momentum toward durable and potentially curative therapeutic strategies. Clinical progress in hemoglobinopathies and hematological malignancies underscores the ability of genome editing to overcome key limitations of conventional treatments, including lifelong symptom management, dependence on donor availability, and risks of disease recurrence or relapse. Simultaneously, innovations in nuclease fidelity, transient expression systems, and comprehensive safety monitoring approaches have strengthened confidence in genomic integrity and long-term clinical feasibility. Responsible integration of CRISPR-Cas9 into advanced care pathways is further supported by robust ethical governance, the maturation of regulatory frameworks, and the expansion of global clinical trial activity. Persistent challenges related to manufacturing scalability, cost, workforce preparedness, and equitable access continue to influence clinical adoption. Addressing these barriers through harmonized regulation, infrastructure development, and long-term outcome surveillance will be critical. Collectively, current evidence positions CRISPR-Cas9 as a cornerstone technology in the future of precision, mechanism-driven hematological therapy.
